# Why Don’t Younger Solicitors Choose Criminal Legal Aid? Insights from the Theory of Planned Behaviour

**DOI:** 10.1007/s10609-026-09528-7

**Published:** 2026-10-14

**Authors:** Susan Elizabeth Rockey

**Affiliations:** Law school, University of Exeter, Exeter, UK

## Abstract

The decline of younger solicitors entering criminal legal aid threatens the sustainability of the criminal defence profession in England and Wales. This study applies the Theory of Planned Behaviour to examine whether commonly cited recruitment barriers influence aspiring solicitors’ decisions not to specialise in this sector after graduation. Survey data from 525 law students under the age of 35 were analysed using Partial Least Squares Structural Equation Modelling. The findings indicate that attitudes and perceived behavioural control significantly predict intentions not to specialise. Negative beliefs relating to remuneration, workload, and student debt were found to shape unfavourable attitudes, while limited familiarity with the profession and restricted access to training opportunities contributed to reduced perceptions of control. Practically, the findings highlight key areas that CLA firms and policymakers may need to address to improve recruitment. The study also extends the Theory of Planned Behaviour into a new professional context, demonstrating the value of indirect measures in explaining career decision-making.

## Introduction

In England and Wales criminal defence solicitors whose firms hold contracts with the Legal Aid Agency to provide legal aid services are commonly referred to as criminal legal aid (CLA) solicitors.^1^ They ensure that individuals have access to free legal assistance at the police station where a duty or own solicitor is requested.^2^ Moreover, they provide advice, assistance, and representation to defendants who satisfy both the means (financial) test and the interests of justice test set out in Section 17(1) of the Legal Aid, Sentencing and Punishment of Offenders Act 2012 (LASPO), extending beyond the police station stage.^3^ Accordingly, CLA solicitors are a key mechanism for supporting access to justice and a vital component of the criminal justice system (CJS) in England and Wales.

Between 2023 and 2024, solicitors providing CLA services in England and Wales dealt with 584,616 cases at police stations, 270,302 matters before magistrates’ courts, and 84,988 in the Crown Court.^4^ Compared with 2020–21, these figures represent increases of 83,222, 43,638, and 17,183 cases respectively.^5^ Such growth is unlikely to be short-lived. The government’s initiative to recruit 20,000 additional police officers between 2020 and 2024 is expected to result in higher arrest rates, thereby sustaining demand for CLA services.^6^ This trend is reinforced by a recent House of Lords briefing on reducing the Crown Court backlog, which reported that active criminal cases, including trials, ongoing matters, and sentencing hearings, reached 73,105 in September 2024, almost double the 38,016 recorded in late 2019.^7^

This sustained rise in demand is occurring alongside a contraction in the number of practitioners able to meet it. Between 2014–15 and 2022–23, the total number of CLA solicitors fell from 14,799 to 10,520, while the duty solicitor pool declined from 4,992 to 3,809.^8^ This reduction is not evenly distributed; it is particularly acute among younger practitioners. Between 2017 and 2023, the number of CLA solicitors under the age of 34 fell by almost 29%, from 3,186 to 2,270.^9^ Among duty solicitors, the decline was even more pronounced, with numbers halving from 694 to 333.^10^

The loss of younger practitioners is especially concerning as they represent the future sustainability of the profession. At present, more than a quarter of CLA solicitors and approximately one-third of duty solicitors are aged 55 or over and nearing retirement.^11^ The insufficient replacement of this cohort is likely to intensify the structural mismatch between supply and demand that is emerging across England and Wales. Evidence from the LexisNexis report suggests that regions including the South West, South East, and East of England are “legal aid deserts”, as they are those most in need of CLA support yet have the least access to it.^12^ Statistics also indicate that 25 out of 189 duty solicitor schemes lack sufficient members to provide full weekly coverage.^13^

The implications of this imbalance raise serious concerns for access to justice, a core component of the CJS, a fundamental aspect of the rule of law, and a right recognised in both the common law and the ECHR.^14^ In particular, it risks undermining the practical effectiveness of Article 6(3)(c) ECHR, which guarantees the right to legal assistance, free of charge where the interests of justice so require, from the initial stages of police interrogation unless compelling reasons justify restriction.^15^

Where legal assistance is unavailable, suspects and defendants may be compelled to represent themselves at the police station or in court. As Harris et al. argue, the absence of representation places individuals at a significant and enduring disadvantage vis-à-vis the state.^16^ The Grand Chamber of the ECHR has similarly emphasised, in *Beuze v Belgium*, that denial of access to a lawyer during police questioning may undermine the principle of equality of arms inherent in Article 6(1), by depriving the accused of a reasonable opportunity to present their case without substantial disadvantage.^17^

One manifestation of this disadvantage lies in the difficulty of understanding procedural rights and evidential implications, particularly during police detention. Empirical research by Newman and Dehaghani, based on interviews with individuals accused of criminal offences, indicates that such information is frequently perceived as confusing and inaccessible.^18^ This is especially significant given the Court’s recognition in *Salduz v Turkey* that suspects are in a particularly vulnerable position at this stage of proceedings.^19^ Moreover, as acknowledged in *Beuze v Belgium*, the lack of access to legal assistance at the pre-trial stage may contribute to miscarriages of justice.^20^ It also, as emphasised in *Salduz v Turkey*, removes a critical safeguard against coercion and ill-treatment and undermines the right against self-incrimination.^21^

The effects of this imbalance could also be felt across the wider CJS. Police investigations may be disrupted where suspects request legal advice but no solicitor is available, increasing the likelihood of release prior to interview.^22^ Similarly, magistrates’ courts may face a growing number of litigants in person, which, as the Magistrates’ Association has noted, can reduce efficiency and further delay proceedings within an already strained system.^23^

In sum, the structural mismatch between supply and demand emerging across England and Wales is likely to intensify if the decline in younger CLA solicitors continues to limit the replacement of those approaching retirement. This would significantly undermine access to justice, particularly the right to a fair trial, placing suspects and defendants at a structural disadvantage and weakening the effective functioning of the CJS as a whole.

## Drivers of the Decline

The Solicitors Regulation Authority reports that the average age of admission to the Roll of Solicitors in 2021–22 was 30, suggesting that the shrinking younger solicitor cohort largely consists of newly qualified solicitors.^24^ As career specialisation choices, particularly in law, are often formed prior to qualification, the decline in newly qualified CLA solicitors is therefore likely linked to decisions made during legal education. Safeguarding access to justice therefore requires an understanding of why fewer law students aspiring to become solicitors (aspiring solicitors) are drawn to CLA, in order to inform effective measures to increase recruitment.

Key reports from stakeholder groups and academic studies provide sector-specific insights into recruitment barriers within CLA practice, derived from investigations into the CLA system and the wider legal aid landscape. The two most comprehensive of these are the 2021 *Independent Criminal Legal Aid Review (CLAIR)*, conducted by former judge Lord Bellamy KC, which examined the CLA system in its entirety, including the services provided and the mechanisms through which they are procured and funded, and the 2021 *Legal Aid Census*, which provides insights into the legal aid landscape from the perspectives of those working day-to-day in the sector.^25^

Across these reports, a common theme emerges: the reductions to CLA fees introduced in 2014 by the Conservative–Liberal Democrat coalition government, together with prolonged fee stagnation, have generated a series of structural barriers to recruitment.^26^

However, following the *CLAIR* additional investment in CLA solicitor fees, amounting to approximately 18%, has been implemented in phases. The first phase, introduced in September and October 2022, delivered an immediate 15% increase to most fees and extended remuneration to all pre-charge engagement work, equating to an overall uplift of around 9%.^27^

The second phase focused on longer-term system reform. In December 2024, a further £24 million was invested in police station and magistrates’ court fee schemes, increasing the total uplift to approximately 12%.^28^ The third phase followed the UK Government’s December 2024 announcement of a further 12% increase in CLA rates.^29^ The first 6% of this additional investment was implemented in December 2025, this included the harmonisation of police station attendance fees, alongside uplifts to prison law fees, magistrates’ court fees (including Youth Court fees), and certain appeal fees.^30^ This was followed in February 2026 by an additional further fee increases and restructuring to the Litigators’ Graduated Fee Scheme, as well as uplifts to remaining areas of appeals work not covered in December 2025.^31^ The remaining 6% is expected to be delivered over the course of the current Parliament.^32^

Drawing on Lord Bellamy QC’s analysis in the *CLAIR* that a 15% increase in fees was required, the 18% increases, suggest, at least on paper, that firms may now have greater capacity to address associated structural barriers to recruitment. However, given the recency of these changes and the current lack of firm-level empirical research to substantiate this in practice, it remains too early to determine how far these reforms have translated, or will translate, into meaningful changes in recruitment. This study therefore proceeds on the basis that the cited structural barriers to recruitment remain relevant, while recognising that their present significance may be in transition.

### Salaries

The most frequently cited structural barrier to recruitment in the literature is the level of salaries that CLA firms are able to offer. As highlighted in the *CLAIR*, the challenge of paying competitive wages from an income derived from fees that have remained effectively frozen since 2008 is described as “inherently a losing battle.”^33^

This perception is also reflected in the views of prospective entrants. Findings from the *CLAIR Student Survey* indicate that over half (53%) of law students identified low pay as a primary deterrent to entering criminal defence work, frequently citing concerns about financial stability and the relative attractiveness of alternative legal careers.^34^ Similarly, Durham University’s *Criminal Law Futures Survey* found that a significant proportion of respondents cited low pay as the principal reason for their lack of interest in criminal practice.^35^ These findings suggest that the salary constraints associated with fee stagnation are not only experienced within the profession but are also clearly understood by those considering entry into it.

The literature further emphasises that the issue is not solely the absolute level of pay, but its relative competitiveness. Salaries offered by the Crown Prosecution Service (CPS), for instance, are consistently higher.^36^ Evidence presented to both the House of Commons Justice Committee and the Westminster Commission indicates that such disparities make these alternative roles within the CJS financially more attractive.^37^ Academic research supports this conclusion: Dr Thornton reports that trainees are increasingly leaving criminal defence for better-paid roles elsewhere, while Dr Kemp similarly finds that individuals initially interested in defence work are pursuing alternative career paths within the sector.^38^

### Qualification and training pathways

Alongside remuneration, the literature identifies access to qualification and training as a significant structural barrier to recruitment. Since September 2021, prospective solicitors must complete both stages of the Solicitors Qualifying Examination (SQE), in addition to a period of qualifying work experience.^39^ The availability of such opportunities within CLA firms is therefore critical for those seeking to enter the field. However, empirical data from the *Legal Aid Census* indicates that 43.8% of legal aid organisations do not offer training opportunities.^40^

The literature consistently links this contraction in training provision to financial pressures arising from fee stagnation. For example, evidence presented to the *CLAIR* includes practitioners reporting that they have ceased offering training contracts altogether, citing the financial unviability of absorbing supervision costs given delayed returns on investment.^41^ Similar concerns have been raised in evidence to the Westminster Commission and the House of Commons Justice Committee.^42^

Unsurprisingly, this reduction in entry routes appears to deter aspiring solicitors. Denvir et al. found that 53.6% of respondents identified the lack of training opportunities as the primary factor discouraging them from pursuing a career in legal aid.^43^

In addition, financial barriers associated with qualification further compound the problem.^44^ Unlike many commercial firms, CLA practices are often unable to cover the costs of the SQE, which range from £4,100 to £17,800.^45^ As a result, aspiring solicitors are frequently required to fund qualification independently, incurring significant additional debt. As Dr Thornton observes, this is particularly problematic in a sector characterised by relatively low salaries, limiting the ability of entrants to offset these costs.^46^

This dynamic has important implications for social mobility. Reports by Young Legal Aid Lawyers highlight that individuals from lower socio-economic backgrounds are more likely to prioritise higher-paying career paths in order to manage debt, reinforcing conclusions drawn by the Westminster Commission that entry into CLA is, in practice, more accessible to those with independent financial means.^47^

Overall, the literature indicates that underfunding not only reduces the availability of training opportunities but also shifts the financial burden of qualification onto entrants, thereby significantly constraining access to the profession.

### Career familiarity and exposure

Beyond financial and structural barriers, the literature suggests that limited familiarity with CLA as a career option may also undermine recruitment. Aspiring solicitors typically develop their understanding of different practice areas through degree modules, extracurricular activities, law fairs, and work experience or vacation schemes, which provide insight into the realities of legal work and facilitate engagement with employers. These experiences are widely recognised as playing a significant role in shaping career aspirations.^48^

However, evidence indicates that such exposure is limited within CLA. Data from the *CLAIR Student Survey* shows that 16% of law students reported a lack of information about CLA careers, while the *Legal Aid Census* similarly identifies gaps in awareness among those considering legal careers more broadly.^49^

This lack of visibility can itself be linked to financial constraints within the sector. Limited fee income restricts firms’ capacity to offer work experience placements, participate in university recruitment initiatives, or maintain a consistent presence in early-career pathways. Furthermore, where firms are unable to provide training opportunities, there is reduced incentive to invest in outreach activities. As a result, underfunding indirectly limits exposure to CLA practice, reducing awareness and interest among aspiring solicitors. In turn, this diminished visibility may function as an additional barrier to recruitment.

### Working conditions and hours

The literature also highlights that working conditions have been impacted by the reduction and prolonged stagnation of CLA fees. Empirical research by Professor Newman suggests that reductions in legal aid fees have significantly altered the economics of practice, with solicitors reporting the need to manage substantially higher caseloads in order to remain financially viable.^50^ One practitioner described undertaking “three times the amount of work” to achieve the same level of income as in previous years, while others emphasise that increasing volume is the only way to offset low fixed fees.^51^

These pressures are further compounded by the long and often unsociable hours associated with CLA practice. For example, solicitors who participate in the duty solicitor rota are required to provide police station representation over a 24-hour period, including weekends.^52^ This adds to an already heavy workload and has been described by the London Criminal Courts Solicitors’ Association as “gruelling,” reflecting the dual demands of daytime office work and overnight duty shifts, often followed by a return to the office the next day.^53^ Such patterns can result in extended working periods of up to 36 hours and make balancing professional and personal commitments particularly difficult.^54^

These working pattens appear to conflict with the strong preference for work-life balance among students. Survey data from UK university students consistently identifies work-life balance as a key career priority, with 91% of respondents in the *Early Careers Survey* rating it as important.^55^ Correspondingly, findings from the *CLAIR Student Survey* indicate that long and unsociable hours are cited by some law students as a reason for not pursuing criminal law careers.^56^

Although the literature does not always explicitly frame these conditions and working hours as recruitment barriers, their implications are clear. A working environment characterised by heavy workload, and long, unsociable hours, especially when combined with low salaries, is likely to diminish the attractiveness of the profession to aspiring solicitors.

## Summary

In sum, the literature presents a consistent and coherent account of recruitment barriers within CLA firms, rooted in the reduction and long-term stagnation of fees. These barriers include low salaries, reduced training capacity, financial barriers to qualification, limited familiarity with the specialism, and demanding workloads coupled with long working hours.

That said, the extent to which these structural recruitment barriers influence aspiring solicitors’ decisions not to specialise in CLA remains unclear. This uncertainty stems from three key limitations.

First, the literature demonstrates limited engagement with aspiring solicitors themselves, despite their decision-making being central to the long-term sustainability of CLA provision. Much of the existing research relies on firm-level or practitioner perspectives and also draws on broader law student populations, including those intending to pursue barrister or non-legal career pathways. This limits its relevance to aspiring solicitors specifically and may not fully capture the motivations, preferences, and constraints shaping their decisions.

The second limitation is the literature’s reliance on qualitative data collection methods such as academic roundtables, calls for evidence, focus groups, and case studies, with a relative absence of quantitative analysis. While these approaches have provided valuable, in-depth, sector-specific insights into recruitment barriers within English and Welsh CLA firms, they remain largely descriptive and exploratory. As a result, the findings tend to identify and contextualise recruitment barriers rather than establish robust, generalisable causal relationships between these barriers and recruitment, particularly among aspiring solicitors.

The final limitation is the absence of an explicit theoretical framework. One of the most effective ways to generate evidence on structural recruitment barriers would have been to work within such a framework, as these are grounded in extensive prior research and provide a structured means of identifying and defining key barriers, as well as clarifying how they relate to one another.^57^ A framework would also have aided interpretation: rather than analysing findings in isolation, results could have been situated within a wider body of knowledge, helping to guide expectations and strengthen explanations.^58^ As a result, this body of work lacks analytical depth in fully explaining the barriers and their relationship to recruitment, particularly among aspiring solicitors, and, in turn, limits its ability to provide actionable insights for both CLA firms and policymakers.

As a result, there is a need for theoretically informed, quantitative research to establish whether relationships exist between these structural recruitment barriers and aspiring solicitors’ decisions not to specialise in CLA in England and Wales after graduation. Without a clearer understanding of these dynamics, there is a risk that the decline may persist, with potential implications for access to justice. Moreover, without such evidence, the firm-level empirical research required to assess the effectiveness of recent reforms to CLA funding, particularly whether they have translated, or will translate, into an improved capacity for firms to address the structural recruitment barriers contributing to the decline in younger solicitors, cannot be meaningfully undertaken.

## Research Aim, Approach and Contribution

In light of the need to establish whether relationships exist between structural recruitment barriers identified in the existing literature and aspiring solicitors’ decisions not to specialise in CLA in England and Wales, this study examines the extent to which these barriers influence those decisions following graduation.

It aims to move beyond perceptions and assess the measurable influence of recruitment barriers on younger solicitors’ career decisions, thereby providing a stronger evidence base for policymakers, professional bodies, and CLA firms seeking to understand the decline in the number of younger solicitors. This will be achieved through the use of quantitative methods. In addition to being grounded in a theoretical framework, to provide a structured means of addressing the research question and interpreting the results.

To the best of the author’s knowledge, this is the first study to focus specifically on aspiring solicitors’ decisions to specialise in CLA, the recruitment barriers within CLA, and the decline in younger solicitors, rather than relying on insights derived from broader investigations into the CLA system and the wider legal aid landscape. It is also among the first to adopt a quantitative approach combined with a theoretical lens in this area. As such, it not only addresses an important gap in the literature with potential implications for access to justice but also contributes to broader sector-specific discussions within CLA and legal aid research, as well as stakeholder understanding.

## Theoretical Foundations: The Theory of Planned Behaviour

As this study seeks to confirm which, if any, of recruitment barriers cited within the current literature influence aspiring solicitors’ decisions not to specialise in CLA, a theoretical framework capable of explaining decision-making is required. In psychology, decision-making is understood as a form of human behaviour, involving cognitive processes through which individuals evaluate alternatives and select courses of action based on their beliefs, values, and preferences.^59^ This extends to career decision-making, where individuals assess different pathways, such as choices of legal specialism.^60^

These cognitive processes are examined within behavioural science frameworks, which integrate insights from psychology, sociology, economics, and related disciplines to explain how decisions are formed.^61^ One such framework is Icek Ajzen’s Theory of Planned Behaviour (TPB), which offers a means of analysing whether the recruitment barriers may be internalised into aspiring solicitors’ attitudes, perceived social pressures, and perceived behavioural control, and how these factors shape career intentions and decisions regarding specialisation in CLA.^62^

While no studies have examined its application to aspiring solicitors in England and Wales, or to decisions not to specialise within the legal profession, a small but established body of research has demonstrated TPB’s suitability for analysing career-related behaviours among university students. For example, Hickerson et al. examined pharmacy students’ intentions to apply for postgraduate residencies, while Solikhah investigated accounting students’ intentions to pursue careers in public accounting.^63^

Accordingly, TPB provides a theoretically grounded and coherent approach for examining whether, and how, recruitment barriers influence aspiring solicitors’ decisions not to specialise in CLA and will therefore be applied in this study. In doing so, this research contributes not only to the criminal justice, legal aid, and legal recruitment literature, but also extends the application of TPB to a previously unexplored professional and geographical context.

### The Theory of Planned Behaviour

**Fig. 1:**
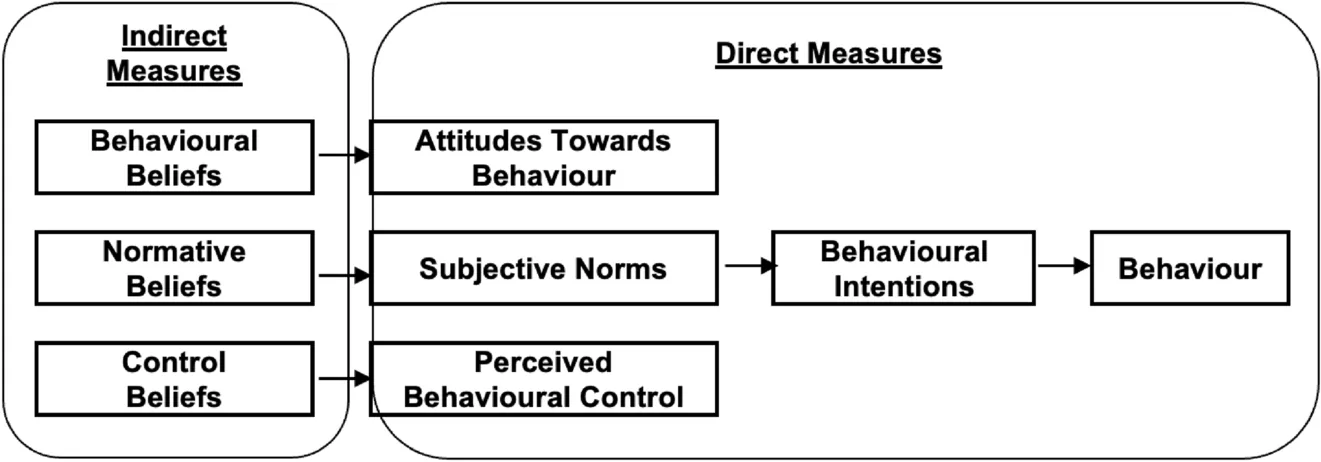
The theory of planned behaviour model

As shown in Fig. 1, the theory has two levels. The first level comprises direct measures, which include intentions, attitudes, subjective norms, and perceived behavioural control. These are the factors that directly shape behaviour. The second level incorporates indirect measures*.* These are the underlying beliefs, behavioural, normative, and control beliefs, that explain why someone holds the direct measures.^64^ For example, aspiring solicitors may think that specialising in CLA after graduation is difficult (an attitude towards specialising) because doing so will lead to low salaries (a behavioural belief) that will make it challenging for them to pursue.

#### Direct measures

##### Intentions

Intentions indicate how much effort individuals are willing to put into carrying out a behaviour.^65^ In simple terms, intentions are the most direct predictor of whether individuals actually perform a behaviour or not.^66^

Many studies have confirmed this link. For example, research found that homeowners in India who intended to build eco-friendly houses were much more likely to follow through.^67^ Similarly, people who said they intended to buy organic milk were more likely to actually purchase it.^68^

##### Attitudes, social norms, and perceived behavioural control

As shown in Fig. 1, three factors, attitudes, social norms, and perceived behavioural control, influence whether someone forms an intention to act.^69^

Attitudes towards a behaviour (ATB) reflects individuals overall favourable, or unfavourable evaluations, or appraisals of the behaviour in question.^70^ A person will have favourable attitudes if they believe a behaviour is likely produce a positive outcome, whereas they will have unfavourable attitudes if they believe it will result in a negative outcome.^71^ For instance, aspiring solicitors who believe specialising in CLA is likely to result in negative outcomes will have unfavourable attitudes.

Subjective norms (SN) refers to the ‘social pressure to perform a behaviour, or not’ individuals perceive.^72^ If aspiring solicitors perceived pressure to perform the behaviour of specialising in CLA this will influence their intention to do so, while perceptions of pressure not to specialise in CLA will lead to intention not to engage in the behaviour.^73^ However, given the social nature of this variable, it will not capture the recruitment barriers that are central to this paper’s aim. As such, this variable is not suitable for inclusion in the theoretical model of this study.

Perceived behavioural control (PBC) reflects how easy, or difficult individuals think it will be to perform the behaviour.^74^ If they perceive a behaviour such as specialising in CLA as being easy to perform, they are more likely to intend to do it, whereas if they expect it will be difficult, their intention is likely to weaken.^75^

A growing body of research has used ATB, and PBC to predict intentions across areas such as health, leisure, consumer choices, and careers. However, no studies have yet examined the career intentions of aspiring solicitors in England, and Wales, making this application of the TPB a novel contribution.

Nevertheless, research on university students in other countries shows that both ATB, and PBC are consistent positive predictors of career-related intentions. For example, Fort et al. found these two direct measures significantly influenced job-searching intentions among jobseekers in France, while Solikhah reported similar results for accounting students in Indonesia.^76^ Overall, these findings support using the TPB framework to study students’ career intentions, and behaviours. Therefore, the following hypotheses are proposed:

###### H1

Aspiring solicitors’ attitudes towards specialising in CLA will be significantly related to their intentions to specialise in CLA.

###### H2

Aspiring solicitors’ perceived control over specialising in CLA will be significantly related to their intentions to specialise in CLA.

#### Indirect measures

Fishbein, and Ajzen described three indirect measures (see Fig. 1) that explain the direct measures.^77^ Behavioural beliefs refer to a population's subjective assessment of the likelihood that performing a particular behaviour will lead to specific outcomes within a defined timeframe, ie. what aspiring solicitors’ think will happen if they specialise in CLA.^78^ Control beliefs concern a population’s subjective perceptions of whether the necessary resources, and opportunities will be available within the timeframe for performing a given behaviour, e.g. what aspiring solicitors’ perceive about whether they will have the resources, and opportunities to carry out the behaviour.^79^

These beliefs are often analysed using Fishbein, and Ajzen’s expectancy-value model (EVM).^80^ This model combines two elements: belief likelihood judgements (expectancy), and belief evaluation judgements (value).^81^ Multiplying these elements for each belief provides an indication of how strongly that belief influences behaviour.

For behavioural beliefs, the specific elements are how likely a particular outcome will be if a behaviour is enacted (belief strength), and is that outcome good, or bad (outcome evaluation).^82^ For control beliefs, the elements focus is on perceptions of whether resources, and opportunities will be present, or absent if the behaviour is performed (belief strength), and the extent to which these perceptions will facilitate, or hinder the performance of the behaviour* (*power of control).^83^

Empirical support for the TPB’s proposition that behavioural, and control beliefs explain why certain attitudes, and perceptions of control in the career context are limited, largely because many studies have not followed Ajzen’s recommendation to use both direct, and indirect measures.^84^ Evidence from non-career contexts, however, supports these links. For instance, Mak, and Davis applied the TPB to study intentions to seek mental health services in Macao, Bhatti, and Husin examined intentions to participate in a family Takaful scheme in the UAE, and Perri et al. investigated intentions to adopt smart consumption, and production behaviours in Italy. All three studies found that behavioural beliefs significantly predicted ATB, and control beliefs significantly predicted PBC.^85^

Therefore, the following hypotheses are proposed:

##### H3

Aspiring solicitors’ behavioural beliefs will be significantly related to their attitudes towards specialising as a CLA solicitor.

##### H4

Aspiring solicitors’ control beliefs will be significantly related to their perceived behavioural control over specialising as a CLA solicitor.

## Methods

The application of the TPB requires two studies: a pilot exploration of beliefs followed by an evaluation.^86^ As outlined below, this study adopts a fully quantitative approach at both stages of data collection, diverging from the TPB’s typical combination of qualitative belief elicitation and subsequent quantitative evaluation.^87^ By doing so, it moves beyond prior research that has primarily identified and contextualised recruitment barriers, enabling a statistical analysis of generalisable relationships between these barriers and aspiring solicitors’ decisions not to specialise in CLA. Specifically, this approach allows for the testing of hypothesised relationships between beliefs about the recruitment barriers, attitudes, and perceived behavioural control, as well as their influence on such decisions.

### The pilot study

Following Ajzen’s guidelines, the pilot exploration aimed to identify behavioural, and control beliefs associated with performing the behaviour under study within a particular population.^88^ This study pilot is discussed in detail elsewhere, where a novel quantitative approach was adopted to identify the beliefs of aspiring solicitors.^89^

The findings indicated that the key behavioural beliefs associated with specialising in CLA included: a trainee salary of £20,000–£25,000; a qualified salary of £25,000–£30,000; salary in relation to workload; salary in relation to working hours; and the prospect of long, and unsociable hours. The control beliefs were: lack of familiarity with the career; absence, or low volume of training opportunities; concerns about additional student debt from self-funding the SQE; and lack of financial support.^90^

These beliefs were then converted into survey items for the quantitative evaluation study, which is the focus of this paper. Specifically, items measured both the strength of a belief (b_i_), and the evaluation of its outcome (e_i_). For example: ‘Having a salary while training that is lower than a trainee Crown Prosecutor will receive for a similar role*’* (belief strength), paired with ‘For me, having a salary while training that is lower than a trainee Crown Prosecutor will receive for a similar role is unlikely _ _ _ _ _ likely’ (evaluation of that outcome). Similarly, two sets of items were developed to measure the strength of control beliefs (c_i_), and perceptions of control (p_i_). For instance: ‘Whether criminal defence firms hire graduates’ (belief strength), and ‘Not having the opportunity to become familiar with whether criminal defence firms hire graduates will make it easier _ _ _ _ _ more difficult to specialise as a CLA solicitor’ (power of control).

### Quantitative evaluation questionnaire

The online, self-completed quantitative evaluation questionnaire consisted of five sections, and was designed to test hypotheses H1–H4 by examining all direct, and indirect TPB variables. It was developed on Qualtrics, following the guidelines set out in the manual for constructing TPB questionnaires.^91^ Ethical approval was obtained from the University of Exeter’s Research Ethics Committee (reference: 1212432).

Section One established participant eligibility with three screening questions: (1) Are you a law student at a university in England, or Wales? (2) Do you aspire to be a solicitor? (3) Are you aged 35 years, or under? Eligible participants proceeded to Section Two, which presented the participant information sheet, and sought informed consent.

Section Three gathered demographic information using UK government census categories, including age, gender, degree level, university attended, family social class, and ethnic group.

Section Four included eleven questions designed to measure the TPB variables. Questions One, and Two measured behavioural belief strength (b*i*), and outcome evaluation (e*i*). Participants first rated whether each belief item would apply to them if they specialised in CLA after graduation, using a seven-point unipolar semantic differential scale (1–7). They then evaluated the outcomes on a bipolar scale ranging from – 3 to + 3.

Questions Three to Five assessed control beliefs. Question Three asked how likely it was that, by graduation, participants would not have had the opportunity to become familiar with career-related items (e.g., ‘how to become a criminal defence solicitor through work experience’). Question Four asked how likely it was that the two listed training barriers would be present. Both were rated on seven-point unipolar scales. Question Five measured the power of control (p*i*): participants indicated, on a seven-point bipolar scale, whether each of the five listed items (e.g., lack of relevant work experience) would make it easier, or harder to specialise in CLA.

Question Six measured attitudes towards specialising in CLA, asking participants to rate the career using four pairs of opposite adjectives (e.g., extremely unpleasant – extremely pleasant) on seven-point unipolar scales.

Perceived behavioural control was assessed in questions Seven to Ten, each rated on seven-point unipolar scales with varying endpoints. For example: ‘I am confident that I could specialise as a criminal defence solicitor in England, and Wales after graduation if I wanted to,’ rated from strongly disagree (1) to strongly agree (7).

Question Eleven captured intention not to specialise. Participants rated five intention items, such as ‘I intend to specialise as a criminal defence solicitor in England, and Wales after graduation,’ on a seven-point unipolar scale (1= strongly disagree; 7= strongly agree).

Finally, Section Five offered participants the chance to enter a prize draw for one of four £50 Amazon vouchers. To preserve anonymity, a separate Qualtrics link was provided for email submissions.

Following completion of the survey design, but before data collection, a pilot test was conducted to assess clarity, length, and redundancy of items, using evaluation questions adapted from Francis et al.’s *Constructing Questionnaires Based on the TPB Manual*.^92^ For example, participants were asked whether any items, or instructions were ambiguous, and whether there was anything else the investigators should know about the instrument. 56 participants, recruited via social media, completed the pilot in line with Connelly’s recommendation of 10% of the main sample size (see below).^93^ Feedback indicated that the items, and instructions were clear, and that the survey was not overly long. The main issue identified was difficulty responding on mobile devices due to the answer scale format, which was resolved by adopting a more mobile-friendly design verified through Qualtrics’ preview function.

### Participants

The research population consisted of individuals meeting this paper’s aspiring solicitor criteria. As it was not possible to survey the entire population, a sample size sufficient to ensure meaningful results was calculated. The required sample size was determined using Soper’s *Priori Sample Size Calculator* in four stages.^94^ First, the expected strength of the hypothesised relationships (effect size) was set to medium (0.5), following standard social science guidance, and prior TPB research.^95^ Second, the number of observed items (24), and latent variables (5) in the model were entered.^96^ Third, the significance level was set at 0.05, indicating a 5% probability that results could occur by chance.^97^ Finally, the desired statistical power was set at 0.80, meaning that if a true effect exists, there was an 80% probability of detecting it.^98^ This process indicated a minimum required sample size of 92.

To recruit the required 92 participants, a voluntary response sampling technique was employed between November 2023, and March 2024. Participants were initially recruited via their law schools, and lecturers, ensuring that all eligible individuals had the opportunity to participate. Subsequently, social media platforms such as Facebook, X, and LinkedIn were used to promote the survey with a poster.

### Data Analysis

#### Data Preparation

To prepare the survey data for statistical analysis, it was exported from Qualtrics into SPSS (version 29) and checked for any missing values. Next, demographic responses were converted into numerical codes so that the software could process them; for example, ‘one’ was assigned to female, ‘2’ to male, and so on. The PBC_3,_ and PBC_4_ items with reversed scoring (ie., one indicated a positive response, and seven a negative response) were then adjusted so that, consistently across all items, higher numbers indicated more positive responses. Finally, composite scores were created for related items.

Behavioural, and control belief scores were calculated by multiplying the two relevant components (b*i *× e*i* and c*i *× p*i*). Deviating from the EVM equation, all composites *i*_5_ were combined into single measures (BB_1_–BB_4_ and CB_1_–CB_2_), as shown in Table 1.

**Table 1:** Variable transformation illustration

Calculations	Items	New indirect measures
(*bi*_1_ x *ei*_1_) + (*bi*_2_ x *ei*_2_)	BB_1_	A salary while training, and upon qualification that is lower than a trainee Crown Prosecutor will receive for a similar role.
(*bi*_3_ x *ei*_3_) + (*bi*_4_ x *ei*_4_)	BB_2_	A salary that will not reflect the workload, and hours worked.
(*bi*_5_ x *ei*_5_)	BB_3_	A salary that will not allow for repayment of student loans.
(*bi*_6_ x *ei*_6_) + (*bi*_7_ x *ei*_7_)	BB_4_	Working long, and some unsociable hours.
(*ci*_1_ x *pi*_1_) + (*ci*_2_ x *pi*_2_) + (*ci*_3_ x *pi*_3_)	CB_1_	Levels of familiarity with how to become a criminal defence solicitor, what the career is like day-to-day, and whether CLA firms hire graduates.
(*ci*_4_ x *pi*_4_) + (*ci*_5_ x *pi*_5_)	CB_2_	No available training opportunities, or funded LPC/SQE qualifications, and Professional Skills course fees.

#### Descriptive Statistics

Once the data had been prepared, descriptive statistics were generated for the TPB variables to summarise the characteristics of the responses. These statistics included: frequencies, and percentages; the mean response for each variable; and measures of variability, using standard deviations (*SDs*), which indicate how far respondents’ beliefs, attitudes, perceptions of control, and behavioural intentions deviate from the mean.

#### Testing Hypothesis H1 to H4 using PLS-SEM

Although useful, these descriptive statistics did not allow for hypothesis testing. Therefore, a composite-based analysis,known as Partial Least Squares Structural Equation Modelling (PLS-SEM), was adopted to specify, estimate, and evaluate the measurement, and structural model using SmartPLS software.^99^

Unlike multiple regression and correlation analysis, PLS-SEM enables the simultaneous estimation of relationships between latent constructs (TPB variables) and the individual indicators (survey items) that form them.^100^ This allows for a more nuanced analysis of how specific beliefs about recruitment barriers contribute to aspiring solicitors’ decisions, rather than treating these beliefs as aggregated. Moreover, by explicitly modelling measurement error, PLS-SEM provides more robust estimates compared to traditional techniques that assume survey items were perfectly measured.^101^ Additionally, the visual path models and accessible output tables produced by PLS-SEM enhance the interpretability of findings for practitioners and policymakers who may not be familiar with complex statistical methods. Consequently, it is well suited to testing the hypothesised relationships in this study and assessing the extent to which recruitment barriers influence aspiring solicitors’ decisions.

##### Specification of the Measurement and Structural Model

**Fig. 2:**
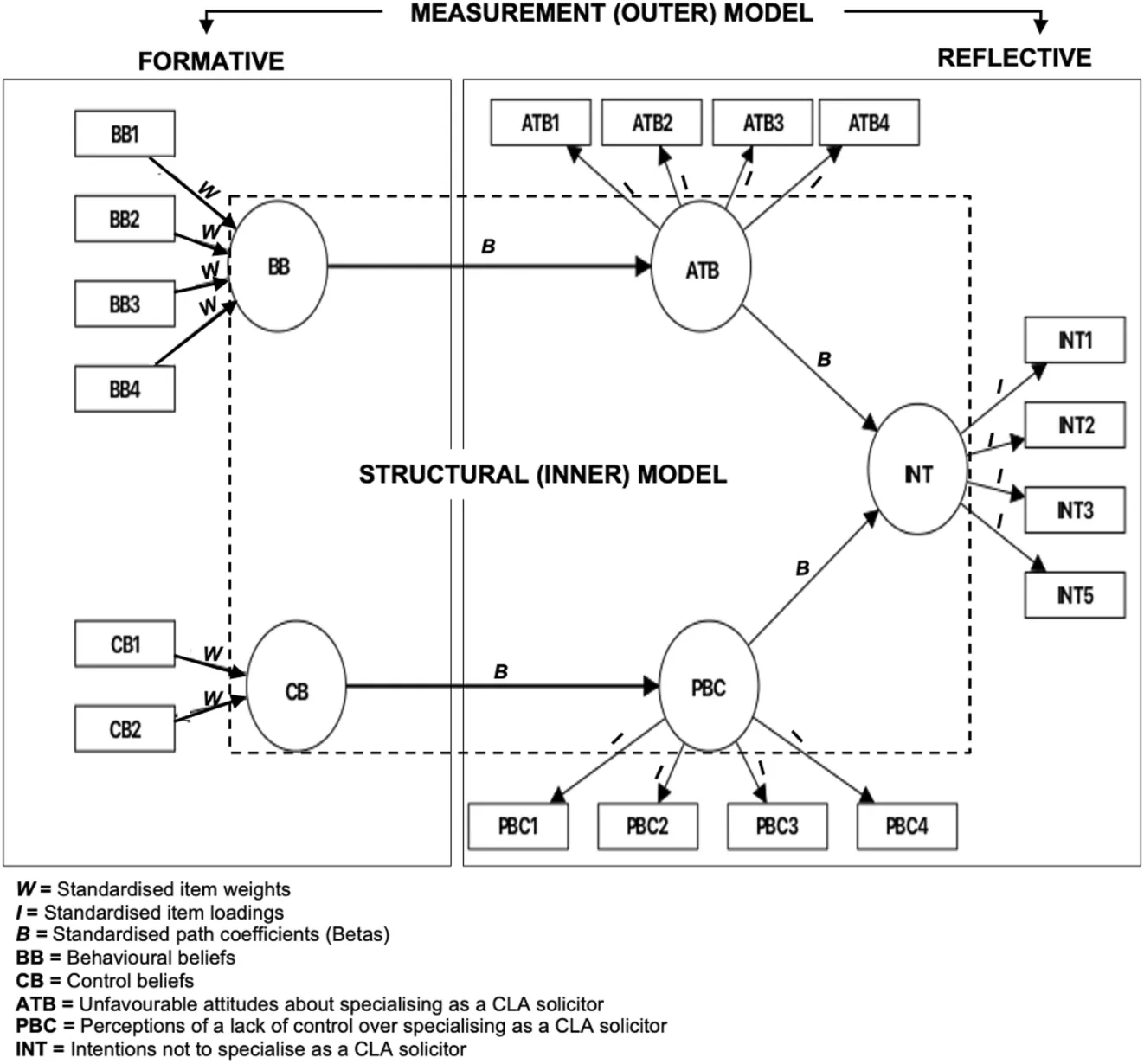
Measurement and structural model

Specification of my measurement model (also referred to as the outer model), and structural model (also known as the inner model) involved using the SmartPLS software to turn the theoretical model (Fig. 1) into the path diagram depicted in Fig. 2.^102^ In this diagram, the key variables, behavioural beliefs (BB), control beliefs (CB), attitudes toward the behaviour (ATB), perceived behavioural control (PBC), and intentions (INT), were represented as circles, while the survey items used to measure them were represented as rectangles.

For the measurement model the arrows (paths) showed how the variables, and items were connected. For reflective variables (ATB, PBC, and INT), the arrows pointed from the variable to the survey items, indicating that the items reflected the underlying variable. For the formative variables (BB and CB), arrows pointed from the items to the variable, showing that the items together formed the variable. In terms of the structural model the arrows represented the hypothesised relationships between the variables, preparing the model for testing the study’s hypotheses.

##### Estimation of the Measurement and Structural Models

The PLS-SEM algorithm was used to calculate estimates for all the paths in the model. This produced standardised item weights (*W*), showing the relative importance of each item in forming the variable, as well as standardised item loadings (*I*), representing the relative contribution of each reflective item to its corresponding variable.^103^ The algorithm also used the total scores for each variable to estimate the hypothesised relationships between variables (H1–H4), producing standardised path coefficients (β) for these relationships.^104^

##### Evaluation of the Measurement and Structural Model

The resulting estimates (β) for the measurement model were evaluated to determine whether the variables items were reliable, valid, and theoretically sound, confirming their suitability for inclusion in the path model. This evaluation was conducted using the following metrics from Henseler, and Dijkstra’s Confirmatory Composite Analysis (CCA), which allowed both reflective, and formative measurement approaches to be assessed.^105^

1. **Item reliability**: For reflective variables, the contribution of each item to its latent variable was assessed using standardised item loadings (I). Items were considered reliable if they explained a sufficient proportion of variance in their variable (> 70).^106^
2. **Internal consistency**: The items for each reflective variable were tested to ensure they consistently measured the same underlying concept, using composite reliability (CR) values. Values within the acceptable range (0.60-0.70) indicated that the items reliably measured the intended construct.
3. **Convergent validity**: This assessed whether the items for a reflective variable captured the same underlying concept.^107^ At least 50% of the variance in the items had to be explained by the variable, as measured by the average variance extracted (AVE) values generated by the PLS-SEM algorithm.^108^
4. **Discriminant validity**: This ensured that different variables were distinct from each other.^109^ The heterotrait-monotrait ratio (HTMT) was used, comparing correlations between items of different constructs to confirm they were sufficiently separate.^110^ Bootstrapping with 10,000 resamples provided confidence intervals to check whether the HTMT values fell below the recommended threshold of 0.85.^111^
5. **Formative items and multicollinearity**: For the formative variables, it was important that each item made a unique contribution. Variance inflation factor (VIF) values were compared against the <3.0 threshold to detect items that were too highly correlated, which could distort results.^112^
6. **Size and significance of item weights**: For formative items, standardised weights (ranging from –1 to 1) were examined to determine each item’s importance to the variable.^113^ Bootstrapping was used to test whether these weights were statistically significant.^114^ This involved comparing the *t-*values from the two-tailed bootstrapping procedure (*t* ≥ 1.96; *t* ≥ 2.58; *t* ≥ 3.291) to the critical *t-*values in the standard normal *t-*distribution table (0.05; 0.01; 0.001).^115^

##### Evaluation of the Structural Model

In terms of the structural model, the evaluation focused on the strength, direction, and significance of relationships, based on the CCA criteria of multicollinearity; size, and significance; *R*^2^; *f*^*2*^ effect size, and the PLSpredict algorithm.^116^ First, the model was checked for collinearity, that is, whether the estimated path coefficients were biased by strong correlations between the predictor variables (ATB and PBC).^117^ This was assessed using the generated VIF values against the <3 threshold.^118^

Next, the size, and significance of the relationships between variables were evaluated using the standardised coefficients (β).^119^ A two-tailed bootstrapping test was also conducted to confirm whether these results were statistically meaningful when compared to the *t-*distribution table values of *t *≥ 1.28;* t *≥ 1.65;* t *≥ 2.33.^120^

The explanatory power of the model was evaluated using the *R*^2^*,* and *f*^2^ effect size metrics. *R*^2^ values indicated how much of the variation in one variable could be explained by others (e.g., how much attitudes, and perceived control explained intentions).^121^ Higher values (> 0.75) reflected substantial explanatory power, values between 0.50, and 0.75 indicated moderate power, and values below 0.25 suggested weak power.^122^ The *f*^2^ values measured how much the ATB, and PBC variables contributed to explaining the INT variable.^123^ Larger values (>0.35) reflected strong explanatory power, values between 0.15, and 0.35 indicated moderate power, and values between 0.02, and 0.15 suggested weak power.^124^

Finally, the model’s predictive power was tested using the PLSpredict algorithm. The dataset was divided into ten equally sized subsets (k-folds), with nine combined into a training sample to predict the remaining subset (holdout sample).^125^ This process was repeated ten times so that each subset served as the holdout once, producing a *Q*^2^ predict statistic.^126^ Once the statistic was confirmed to be greater than zero, the model’s predictive accuracy was evaluated by comparing the PLS-SEM RMSE values with those from a linear regression (LM) benchmark for the INT variable.^127^ If all RMSE values were lower than the LM benchmark, the model demonstrated strong predictive capability.^128^ If most, or an equal number were lower, the capability was moderate; if only a few were lower, it was weak.^129^ Alternatively, if the PLS-SEM RMSE values were higher than the LM benchmark, the model was deemed to have poor predictive capability.^130^

## Results

### Demographic Profile of the Respondents

A total of 525 valid responses were collected, substantially surpassing the minimum required sample size of 92. Participants were aged between 18, and 35, in line with this study’s target profile, with the majority (n = 471, 89.7%) falling within the 18–24 age group. Only 34 respondents (10.3%) were aged 25 to 35.

Most respondents identified as female (n = 385, 73.3%), followed by male participants (n = 139, 26.5%), with one individual (0.2%) reporting a different gender identity. In terms of social class, the largest groups self-identified as working class (n = 239, 45.5%), and middle class (n = 175, 33.3%). Smaller proportions came from the lower class (n = 30, 5.7%), upper class (n = 8, 1.5%), and upper-middle class (n = 73, 13.9%). Respondents represented a diverse set of ethnic backgrounds. The largest category was English, Welsh, Scottish, Northern Irish, or British (n = 243, 46.3%). Other notable groups included Indian (n = 33, 6.3%), Pakistani (n = 30, 5.7%), African (n = 42, 8%), and mixed/multiple ethnic backgrounds (n = 37, 7%).

Academically, the majority of participants were undergraduate law students (n = 459, 87.4%), with the most common programmes being the LLB Law (n = 368, 70.1%), and LLB with Criminology (n = 21, 4.0%). Among postgraduates (n = 63, 12.0%), the LPC (n = 13, 2.5%), and LLM, both with (n = 8, 1.5%), and without (n = 14, 2.7%) SQE preparation, were the most frequent qualifications. Representation from CILEx students (n = 2, 0.4%), and integrated master’s students (n = 1, 0.2%) was minimal. Participants were drawn from 94 of the 109 universities in England, and Wales offering law degrees, with the highest concentrations from institutions in South East England (n = 88, 16.9%) and the Midlands (n = 78, 15%).

Thus, the sample reflected a broad demographic spread. Nonetheless, given that participation was voluntary, it remained possible that aspiring solicitors who chose not to participate differed in some respects from those who did, meaning the results may not entirely capture the characteristics, or perspectives of the wider aspiring solicitor population.

### The TPB variable items

The mean, and *SD* descriptive statistics for newly computed behavioural belief (BB_1_ to BB_4_), and control belief (CB_1_ to CB_2_) composites are presented in Tables 2 and 3.

**Table 2:** Descriptive statistics of the behavioural belief indirect scores

Item	Description	Mean (M)	*SD*
BB_1_	A salary while training, and upon qualification that is lower than a trainee Crown Prosecutor will receive for a similar role.	− 12.01	12.21
BB_2_	A salary that will not reflect the workload, and hours worked.	− 22.80	14.85
BB_3_	A salary that will not allow for repayment of student loans.	− 7.75	7.43
BB_4_	Working long, and some unsociable hours.	− 9.46	16.08

**Table 3:** Descriptive statistics of the control belief composite scores

Item	Description	Mean	*SD*
CB_1_	Levels of familiarity with how to become a criminal defence solicitor, what the career is like day-to-day, and whether CLA firms hire graduates.	− 21.50	18.44
CB_2_	No available training opportunities, or funded LPC/SQE qualifications, and Professional Skills course fees.	− 16.06	12.68

For the belief questions, average scores showed that most participants held negative views about what a career in CLA would involve. In general, they thought that the salaries during training, and after qualification would be lower than those received by trainee, and qualified Crown Prosecutor (BB_1_); the salary would not reflect the workload (BB_2_); the salary would not allow for repayment of student loans (BB_3_); and they would have to work long, and unsociable hours (BB_4_). However, not everyone agreed to the same extent. The large *SD* values for BB_2_ (*SD *= 14.85), and BB_4_ (*SD *= 16.08), indicated considerable variability in the strength of respondents’ negative beliefs, meaning some participants had very strong negative views, while others were less certain, or even neutral.

When it came to beliefs about opportunities, and resources (control beliefs), the picture was similar. On average, respondents believed they lacked familiarity with the sector, and how to enter it (CB_1_), and there would be an absence of training opportunities, and funding for the SQE (CB_2_). Again, with the high *SD* values for these items (*SD *= 18.44, and 12.68) there was a lot of variation. While many saw these issues as barriers, others were more confident that they could still find ways into CLA.

Looking at the bigger picture, the means, and *SDs* for the total scores (ranging from 4 to 28) for the attitudes towards specialising, perceptions of control, and behavioural intentions variables are presented in Table 4. Overall attitudes towards specialising in CLA were positive with a mean score of *M *= 17.32, though their views varied widely (*SD *= 5.33). Some were strongly positive while others were more neutral, or slightly less positive views. In terms of how much control participants felt they had, the average response (*M *= 14.95) suggested a moderate level of control. Most agreed (*SD *= 3.12) they had some ability to specialise in CLA, but they were less confident about this than they were about their general attitudes. Finally, when asked about their intentions to specialise in CLA, the average response (*M *= 12.07) showed only a moderate level of intention. This was the area with the greatest variety in answers (*SD *= 6.54): some respondents had strong intentions to specialise in CLA, while others had weak, or no intentions at all.

**Table 4:** Descriptive statistics of the attitudes, perceptions of control, and behavioural intentions variables

Item	Measures	Mean	*SD*
ATB	Attitudes towards specialising as a CLA in England, and Wales after graduation	17.32	5.33
PBC	Perceptions of control over specialising as a CLA in England, and Wales after graduation	14.95	3.12
INT	Intentions not to specialise as a CLA in England, and Wales after graduation	12.07	6.54

### Measurement Model Evaluations

Table 5 presents the combined results from the evaluation of both the reflective, and formative measurement models. As shown, all model evaluation criteria were met, confirming that the observed items linked to the variables were valid, reliable, and theoretically sound.

**Table 5:** Measurement model evaluation overview

Latent variables	Items	Standardised item loadings and weights (size of loading) 0.40 to > 0.70	CR 0.70–0.95	AVE > 0.50 (50%)	HTMT < 0.85	VIF < 3	Significance < 0.05
ATB (Unfavourable attitudes towards specialising as a CLA solicitor).	ATB_1_	0.843	0.889	0.67	Yes	–	–
	ATB_2_	0.683					
	ATB_3_	0.827					
	ATB_4_	0.900					
PBC (Perceptions of a lack of control over specialising as a CLA solicitor).	PBC_1_	0.864	0.870	0.77	Yes	–	–
	PBC_2_	0.891					
INT (Intentions not to specialise as a CLA solicitor).	INT_1_	0.942	0.939	0.79	Yes	–	–
	INT_2_	0.940					
	INT_3_	0.877					
	INT_4_	0.798					
BB (Behavioural beliefs)	BB_1_	0.477	–	–	–	1.508	Significant at 0.02%
	BB_2_	0.553				1.823	Significant at 0.02%
	BB_3_	0.613				1.432	Significant at 0.001%
	BB_4_	0.516				1.283	Significant at 0.002%
CB (Control beliefs)	CB_1_	0.537	–	–	–	1.452	Significant at 0.05%
	CB_2_	0.595				1.452	Significant at 0.01%

### Structural Model Evaluation

Table 6 presents the results from the evaluation of the structural model. All criteria were met, confirming that the structural relationships between attitudes toward specialising in CLA, perceived behavioural control, and intentions to specialise, were positive, significant, and meaningful. Therefore, with the measurement model also confirming that the observed items linked to the latent variables were valid, reliable, and theoretically sound, the hypotheses were tested.

**Table 6:** Structural model evaluation overview

Latent variable	Items	VIF < 3	Standardised path coefficients (β)	Significance < 0.05 (0.05%)	*R*^2^	*f*^2^	*Q*^2^ >0	PLS__PREDICT_ RMSE < than LM values
ATB	ATB_1_	1.034	0.601	*t* = 0.001	Moderate variation	Large effect size	–	–
	ATB_2_							
	ATB_3_							
	ATB_4_							
PBC	PBC_1_	1.034	0.088	*t* = 0.05	Moderate variation	No effect		
	PBC_2_							
	PBC_3_							
	PBC_4_							
INT	INT_1_	–	–	–	–	–	0.057	No
	INT_2_						0.027	Yes
	INT_3_						0.064	No
	INT_4_						0.010	Yes
BB	BB_1_	–	0.244	*t* = 0.001	Moderate variation	Small effect size	–	–
	BB_2_							
	BB_3_							
	BB_4_							
CB	CB_1_	–	0.223	*t* = 0.001	Moderate variation	Small effect size		
	CB_2_							

### Hypotheses H1 and H2

The analysis showed a strong positive (β = 0.601), and statistically significant (p < 0.001) relationship between attitudes towards specialising in CLA, and the intention to pursue it. Therefore, H1 which postulated that aspiring solicitors’ attitudes towards specialising in CLA in England, and Wales will be significantly related to their intentions to specialise, was supported by the data.

There was also a positive relationship between perceptions of control, and intentions (β = 0.088). Again, this result was statistically significant (p < 0.05), so H2 which posited that aspiring solicitors’ perceptions of control over specialising in CLA in England, and Wales would be significantly related to their intentions to specialise, was also supported by the data.

However, the effect of attitudes (β = 0.601) was far stronger than that of control (β=0.088). This showed that aspiring solicitors’ intentions were shaped more by their overall attitudes than by their perceptions of control.

### Hypotheses H3 and H4

The results also showed a clear positive, and statistically significant relationship between behavioural beliefs, and attitudes (β = 0.244, p < 0.001). As a result, Hypothesis H3, which posited a significant relationship between aspiring solicitors’ behavioural beliefs, and their attitudes towards specialising as CLA solicitors in England, and Wales after graduation, was supported by the data.

In view of this support, the relationships between individual beliefs and attitudes were also examined. The analysis found that a salary while training, and upon qualification that is lower than a trainee Crown Prosecutor will receive for a similar role (BB_1_), a salary that will not reflect the workload, and hours worked (BB_2_); a salary that will not allow for repayment of student loans (BB_3_) and working long, and some unsociable hours (BB_4_) all contributed significantly to this relationship. The standardised loadings for these items were 0.477, 0.553, 0.613, and 0.516 respectively, indicating relatively limited variation in their influence.

There was also a significant relationship between control beliefs, and perceived control (*β *= 0.223, p < 0.001). This meant that hypothesis H4, which posited a significant relationship between aspiring solicitors control beliefs, and their perceived behavioural control over specialising as a CLA solicitor, was also supported.

In light of this support the individual beliefs were again analysed, revealing that both beliefs about the levels of familiarity (CB_1_), and no available training opportunities, or funded LPC/SQE course fees (CB_2_), both contributed significantly to this relationship. With standardised loadings of 0.537 and 0.595, both beliefs again have relatively limited variation in their influence.

## Discussion of Findings

This study set out to confirm whether structural recruitment barriers identified in the literature influence aspiring solicitors’ intentions not to specialise in CLA, using the TPB. The findings provide clear empirical support for this relationship and, in doing so, offer a more robust, generalisable, and analytical explanation for the decline in the number of younger solicitors.

Both attitudes towards specialising in CLA and perceived behavioural control emerged as significant predictors of aspiring solicitors’ intentions not to specialise after graduation. Most notably, attitudes were the dominant predictor, exerting a substantially stronger effect on intentions than perceived control. This indicates that, although perceptions of how difficult it may be to specialise in CLA still influence decisions, these decisions are driven more by how the career is evaluated overall.

The analysis further demonstrates that attitudes are not formed in isolation but are systematically shaped by behavioural beliefs about job attributes. In particular, beliefs relating to low salaries, poor alignment between pay, workload and hours and capacity to repay student loans, and long and unsociable working hours were all influential. The relatively similar weights of these beliefs suggest that no single attribute dominates; rather, negative attitudes reflect a cumulative evaluation of the likelihood that specialising in CLA will lead to these outcomes.

Similarly, aspiring solicitors perceptions of control is shaped by underlying control beliefs. In particular, beliefs about limited opportunities to gain familiarity with CLA, alongside the scarcity of funded training routes, including SQE support, significantly reduced perceived control. As with attitudes, no single belief dominates; instead, perceived control reflects a cumulative assessment of whether the necessary resources and opportunities to enter the profession will be available.

Taken together, these findings suggest that the decline in the number of younger CLA solicitors is  potentially driven by a combination of negative evaluations of the career and perceived constraints on entering the profession. Specifically, aspiring solicitors’ intentions appear to be shaped by expectations of low and uncompetitive salaries, high workload relative to pay, limited ability to repay student debt, and demanding working hours, alongside concerns about limited exposure to the sector and restricted access to funded training pathways.

Given that aspiring solicitors’ negative evaluations play a more decisive role in their decisions, and that recent reforms to CLA fees may take time to translate into firm-level changes, these findings suggest that, if CLA firms are to increase the attractiveness of the profession, priority should be given to addressing these negative perceptions. This includes enhancing salary competitiveness relative to alternative roles such as the CPS, better aligning pay with workload and hours, improving the capacity for early-career debt repayment, and addressing the prevalence of long and unsociable working patterns.

### Implications

These findings have important implications for practice, future research, policy, legal education, and theory.

#### Implications for Practice

For practice, the findings provide theoretically informed, quantitative evidence that structural practices within CLA firms, particularly those relating to pay, workload, training, and familiarity are  potentially contributing to the continued decline in younger solicitors entering the field. This moves beyond prior descriptive accounts in the literature by demonstrating how these factors shape aspiring solicitors’ intentions at the point of career decision-making.

As a result, firms are better positioned to assess whether recent fee increases can be used to remove the recruitment barriers and make these changes. In particular, enhancements salary levels, better alignment of pay with workload, hours and student loan repayment, and the expansion of access to training and funded qualification pathways and opportunities to become familiar with the profession. Without such changes, the current ageing cohort is unlikely to be replaced, which risks exacerbating the structural mismatch between supply and demand identified in the introduction. In turn, this may undermine the practical effectiveness of the right to legal assistance where the interests of justice so require, as well as the broader principle of equality of arms under Article 6(1).

#### Implications for Future Research

The findings also have implications for future research, with downstream relevance for policy and practice in relation to access to justice. By providing a theoretically grounded, quantitative account of how recruitment barriers influence career intentions, this study establishes a baseline against which the impact of recent CLA funding reforms can be evaluated.

   In particular, research, currently in the planning stage, aimed at developing a framework for the recruitment and retention of younger solicitors to support the future sustainability of the CLA workforce in England and Wales. (reference: Susan Rockey, ‘Project Page’ (undated) < https://sites.google.com/view/suzi-rockey/project-page> accessed25September 2026).

#### Implications for policy

Given that the findings indicate decisions not to specialise are influenced by aspiring solicitors familiarly with the profession and career, there may be important implications for the Ministry of Justice, if firms can address the other barriers. In particular, policy interventions aimed at improving familiarity with CLA careers at an early stage, before career intentions are firmly established. For example, policymakers could support targeted communication strategies, developed in collaboration with CLA firms and practitioners, to provide clearer and more accessible information about the nature of the work, career pathways, and opportunities within the sector.

 While such approaches will be explored and evaluated as part of my framework research, evidence from other public sector recruitment contexts suggests that well-targeted recruitment campaigns may play a role in play a role in shaping career perceptions and attracting new entrants.^131^

There may also be important implications for the Ministry of Justice, in terms of further reforms, if firms cannot address these barriers based on the recent fee increases alone, which will also be explored as part of my future research. (Reference: Susan Rockey, ‘Project Page’ (undated) <https://sites.google.com/view/suzi-rockey/project-page> accessed 25 September 2026.)

#### Implications for Legal Education

The findings also indicate an important role for universities and law schools. As noted in the introduction, career decisions are often formed during legal education, and the results show that aspiring solicitors have limited familiarity with CLA, which reduces their perceived ability to enter the field.

Accordingly, greater integration of CLA exposure within legal education may be necessary. This could include more targeted career guidance, as well as initiatives such as a clerking scheme similar to that offered at the University of Kent, which allows law students to assist defence lawyers in Crown Court proceedings.^132^ Such initiatives may help address both limited familiarity and negative perceptions of CLA before career intentions are firmly established. These approaches will again be explored and evaluated as part of my framework research. (Reference: Susan Rockey, ‘Project Page’ (undated) <https://sites.google.com/view/suzi-rockey/project-page> accessed 25 September 2026.)

#### Theoretical implications

Theoretically, this study gives rise to four key implications. First, the results reaffirm the framework’s robustness for explaining career-related behaviours and extends its reach beyond its dominant applications in health psychology, such as exercise and smoking cessation. Demonstrating its utility for career choice strengthens TPB’s position as a valuable framework in vocational and organisational psychology.

Second, by finding that belief-based measures operate as theorised, the study highlights the TPB’s dual strength: not only predicting intentions but also explaining the belief structures that underpin them. This advances understanding of how career-related attitudes and control perceptions are formed.

Third, the study extends the framework into a new professional context by investigating aspiring solicitors’ intentions to specialise in CLA. This demonstrates its potential to illuminate career decision-making across other underexplored professions, opening new avenues for research in vocational and organisational psychology.

Fourth, by applying the model to the English and Welsh context, the study addresses a gap in the literature. Few studies have examined this setting. Confirming the model’s validity here supports its cross-cultural generalisability and underscores its applicability across diverse professional and regional contexts.

### Limitations

While these implications are important, they should be considered in light of several limitations that shape the interpretation of these findings.

**Sample Composition**. The sample consisted primarily of English and Welsh law undergraduate students, with only a small proportion of SQE candidates (2.7%). This limits the generalisability of the findings to the wider population of aspiring solicitors, particularly those entering the profession through non-law undergraduate routes. This exclusion reflects the timing of the study: as the data collection formed part of a PhD thesis, the sampling strategy was determined prior to its commencement. At that time, the Legal Practice Course remained the primary route to qualification as a solicitor, typically requiring either a law degree or a Graduate Diploma in Law. Consequently, the sample reflects the qualification pathways that were dominant at the time of planning and does not include non-law undergraduates who are now able to qualify via the SQE. In hindsight, the sampling strategy should have been revised to reflect the introduction of the SQE route; as a result, the study does not fully capture the diversity of beliefs and intentions within the aspiring solicitor population. Further research should therefore examine a sample of non-law students who aspire to become solicitors, in order to determine whether their attitudes, perceptions, and beliefs influence their decisions in the same way as those of the law students included in this study.

**Sampling Bias**. The study relied on voluntary response sampling, whereby participants self-selected to take part. While this method was valuable for capturing individuals with strong opinions, particularly important for exploring barriers to CLA, it introduces the possibility of self-selection bias. Aspiring solicitors with stronger views on CLA may be overrepresented, while those less invested in the issue may be underrepresented. This potential imbalance could limit the representativeness of the findings, particularly when generalising to the broader solicitor population.

**Exclusion of Subjective Norms**. The TPB framework posits that subjective norms, perceived social pressures to perform, or avoid a behaviour, also predict intentions.^133^ In the context of aspiring solicitors, such pressures may stem from family members, lecturers, career advisors, or practising lawyers. These potential influences were excluded from the model on the grounds that they were not identified as barriers in the existing literature that this study sought to test. In retrospect, their omission may have overlooked important social dynamics that could further inform understanding of the decline in the number of younger solicitors. Excluding subjective norms may therefore limit the model’s capacity to capture the full range of factors influencing career intentions. Further research exploring the relationship between subjective norms and aspiring solicitors’ decisions would extend these findings and provide a more comprehensive understanding of the factors influencing career intentions.

## Conclusion

The decline in younger solicitors specialising in CLA in England and Wales represents a significant challenge to the sustainability of the CJS and risks exacerbating a structural mismatch between the supply of, and demand for, legal services, which may in turn undermine access to justice. In particular, it threatens the practical effectiveness of the right to legal assistance where the interests of justice so require, as well as the broader principle of equality of arms under Article 6(1).

Previous reports have highlighted concerns about recruitment, often attributing these to structural barriers arising from reductions in legal aid funding and prolonged fee stagnation. However, these conclusions have largely been drawn without substantial empirical data from aspiring solicitors themselves, despite their decision-making being central to the long-term sustainability of CLA provision given the current aging profession. Moreover, much of the existing research has relied on qualitative approaches, limiting the ability to establish generalisable relationships between perceived barriers and career intentions. The absence of a clear theoretical framework has also constrained the analytical depth of these studies, particularly in explaining how different barriers shape decision-making.

Against this backdrop, this study employed quantitative methods and the TPB to examine whether the structural recruitment barriers identified in the literature influence aspiring solicitors’ decisions not to specialise in CLA. The findings demonstrate that attitudes and perceived behavioural control are significant predictors of these intentions. Specifically, negative perceptions relating to low salaries, poor alignment between pay, workload, and hours, and concerns about repaying student debt were found to shape unfavourable attitudes. At the same time, limited familiarity with the profession and the scarcity of funded training routes, including SQE support, were found to reduce perceptions of control. Taken together, these findings provide empirical support for the recruitment barriers identified in the literature and offer a more robust, theoretically grounded explanation for the decline in younger solicitors entering CLA.

These findings have important implications for CLA firms, particularly in assessing whether recent fee increases can be used to attract and recruit younger solicitors. There are also implications for the Ministry of Justice, as well as universities and law schools, in relation to career familiarity. Efforts to improve awareness of CLA careers at an early stage, before career intentions are firmly established, may be particularly valuable. This could include initiatives such as clerking schemes, which provide law students with direct exposure to criminal defence practice. More broadly, there may be value in drawing on approaches used in other public sector professions, such as the NHS and UK Civil Service, where coordinated recruitment campaigns have been used to reshape perceptions and attract new entrants. Such initiatives could involve a government funded promotion of the benefits of working in the sector and showcasing the experiences of CLA solicitors. However, further research is required to assess whether such strategies would be effective in enhancing familiarity.

Further implications arise for the Ministry of Justice if CLA firms are unable to address recruitment barriers through recent fee increases alone. In such circumstances, additional structural reform may be required. One potential approach would be the reintroduction of government-funded training grants, covering a proportion of trainee salaries as well as SQE preparation and examination costs. However, research would needed to evaluate the effectiveness of such measures, including their impact on firms’ recruitment capacity and the extent to which aspiring solicitors would take up such opportunities.

Notwithstanding these contributions, this study also highlights several avenues for future research arising from both its limitations and its findings. In particular, research aimed at developing a framework for the recruitment and retention of younger solicitors to support the future sustainability of the CLA workforce in England and Wales. (Reference: Susan Rockey, ‘Project Page’ (undated) <https://sites.google.com/view/suzi-rockey/project-page> accessed 25 September 2026.) There is also scope within this framework to extend the model by incorporating subjective norms, in order to explore the influence of social pressures, such as those from family members, educators, and practitioners, on career decisions. In addtion, to examining the attitudes, perceptions, and beliefs of non-law students who aspire to become solicitors, in order to determine whether the factors identified in this study influence their decisions to specialise in CLA in the same way as law students, and whether recent fee increases translate into meaningful changes at the firm level, and whether such changes are sufficient to improve recruitment capacity.

The findings contribute to practice by highlighting the job attributes shaping aspiring solicitors’ decisions not to specialise in CLA in England and Wales. They also have implications for theory by extending the TPB into a novel professional domain and to policy by highlighting the structural reforms required to make CLA a viable long-term career.

## Data Availability

The research data supporting this publication are provided within this paper.

## Footnotes

1 The Legal Aid Agency, 'Procurement At LAA' (*GOV.UK*, undated) https://www.gov.uk/government/organisations/legal-aid-agency/about/procurement accessed 17 May 2022.

2 Police and Criminal Evidence Act 1984, Section 58.

3 The Legal Aid, Sentencing, and Punishment of Offenders Act 2012, Section 17(1)(a) and Section 17(1)(b); Parliament.UK, ‘Criminal Legal Aid’ (*Parliament.UK,* 26 July 2018) <https://publications.parliament.uk/pa/cm201719/cmselect/cmjust/1069/106905.htm> accessed 15 March 2022.

4 Ministry of Justice, ‘Legal Aid Statistics England and Wales Bulletin Apr to Jun 2024, Tables 2.1 and 4.1’ (*GOV.UK*, 26 September 2024) <www.gov.uk/government/statistics/legal-aid-statistics-quarterly-april-to-june-2024/legal-aid-statistics-england-and-wales-bulletin-apr-to-jun-2024> accessed 25 October 2024.

5 Ibid.

6 GOV.UK, 'National Campaign to Recruit 20,000 Police Officers Launches Today' (*GOV.UK*, 2019) https://www.gov.uk/government/news/national-campaign-to-recruit-20000-police-officers-launches-today accessed 16 November 2021.

7 Claire Brader, ‘In Focus. Reducing the Crown Court Backlog’ (*House of Lords Library*, 13 March 2025) <https://lordslibrary.parliament.uk/reducing-the-crown-court-backlog/> accessed 2 June 2025.

8 Ministry of Justice, *supra* note 4, Table 12.2.

9 Ibid.

10 Ibid; Susan Rockey, ‘The Age Imbalance in the Duty Solicitor Scheme: Exploring the Role of Solicitors Qualifying Examination Costs and Criminal Legal Aid Policy’ [2025] International Journal of the Legal Profession 1, https://doi.org/10.1080/09695958.2025.2551519> accessed 29 September 2025.

11 Ibid.

12 LexisNexis*, The LexisNexis Legal Aid Deserts Report* (LexisNexis 2024) <www.lexisnexis.co.uk/insights/the-lexisnexis-legal-aid-deserts-report/index.html#group-section-Methodology-LBdWRNAE5P> accessed 13 September 2024.

13 Ministry of Justice, *supra* note 4, Table 9.8.

14 Equality and Human Rights Commission, *Following Grenfell: Access to Justice*(Equality and Human Rights Commission 2019) <www.equalityhumanrights.com/sites/default/files/following-grenfell-briefing-access-to-justice.pdf> accessed 27 September 2024.; *R v Lord Chancellor ex p Witham* [1998] QB 578; Ministry of Justice, *Legal Support: The Way Ahead* (Ministry of Justice 2019) <https://assets.publishing.service.gov.uk/media/5c5b3a0840f0b676e6ddc6dc/legal-support-the-way-ahead.pdf> accessed 27 September 2024.

15 *Salduz v Turkey* App no 36391/02 (ECtHR, 27 November 2008) [55].

16 Nicola Harris, Roxanna Dehaghani and Daniel Newman, ‘Vulnerability, The Future of The Criminal Defence Profession, and The Implications for Teaching and Learning’ (2021) 55(1) The Law Teacher 57 http://dx.doi.org/10.1080/03069400.2021.1872872> accessed 15 July 2023.

17 *Beuze v Belgium* App no 71409/10 (ECtHR, 9 November 2018) [125].

18 Newman Daniel and Dehaghani Roxanna, *‘Navigating the Criminal Justice System: Introduction’* in Newman Daniel and Dehaghani Roxanna, Experiences of Criminal Justice: Perspectives from Wales on a System in Crisis (Policy Press 2022) <http://dx.doi.org/10.1332/policypress/9781529214222.003.0007> accessed 26 July 2023.

19 *Salduz v Turkey* App no 36391/02 (ECtHR, 27 November 2008) [55].

20 *Supra* note 17.

21 *Supra* note 19.

22 The Law Society, ‘Perfect Storm for Policing, Law and Order by 2027’ (*The Law Society,* 14 June 2023) <www.lawsociety.org.uk/topics/research/duty-solicitor-projections> accessed 26 July 2023.

23 Magistrates Association, ‘Written Evidence from the Magistrates Association’ (*Parliament.UK,* undated) <https://committees.parliament.uk/writtenevidence/12950/html/#_ftn1> accessed 11 September 2023.

24 *Supra* note 22.

25 Lord Christopher Bellamy KC, *Independent Review of Criminal Legal Aid* (29 November 2021) https://assets.publishing.service.gov.uk/government/uploads/system/uploads/attachment_data/file/1041117/clar-independent-review-report-2021.pdf accessed 6 January 2022; Catrina Denvir and Others, *We Are Legal Aid Findings From the 2021 Legal Aid Census* (Legal Aid Practitioners Group 2022) <https://lapg.co.uk/wp-content/uploads/We-Are-Legal-Aid_Findings-from-the-2021-Legal-Aid-Census_Final.pdf> accessed 12 June 2023.

26 Lord Christopher Bellamy KC, *supra* note 25; Owen Bowcott, 'Legal Aid: How Has It Changed In 70 Years?' (*The Guardian*, 2018) https://www.theguardian.com/law/2018/dec/26/legal-aid-how-has-it-changed-in-70-years accessed 24 December 2021.

27 Ministry of Justice, ‘Criminal Legal Aid: Proposals for Solicitor Fee Scheme Reform’ (*GOV.UK*, 9 May 2025) <www.gov.uk/government/consultations/criminal-legal-aid-proposals-for-solicitor-fee-scheme-reform/criminal-legal-aid-proposals-for-solicitor-fee-scheme-reform> accessed 25 September 2025.

28 Ibid.

29 Ibid.

30 Sarah Sackman KC MP Minister Of State, ‘Criminal Legal Aid Solicitor Fees Consultation Response’ (6 February 2026) <https://committees.parliament.uk/publications/51581/documents/285952/default/#:~:text=In%20December%202025,%20I%20wrote,into%20force%20on%203%20March> accessed 18 March 2026.

31 Ibid.

32 Gazette Reporter, Criminal Legal Aid Rates Rise by Further 12%’ (*Law Gazette*, 20 December 2024) <www.lawgazette.co.uk/news/criminal-legal-aid-rates-rise-by-further-12/5121875.article> accessed 14 January 2025.

33 Lord Christopher Bellamy KC, *supra* note 25, para. 6.39.

34 Lord Christopher Bellamy KC, *Annex’s to The Independent Review of Criminal Legal Aid* (29 November 2021) https://assets.publishing.service.gov.uk/government/uploads/system/uploads/attachment_data/file/1041116/clar-independent-review-2021-annexes.pdf accessed 6 January 2022.

35 *Criminal Law Futures Survey* (Durham University) undated; The Law Society, ‘Submission to The Independent Criminal Legal Aid Review’ (*The Law Society,* undated) https://www.lawsociety.org.uk/campaigns/consultation-responses/independent-criminal-legal-aid-review-law-society-response accessed 8 February 2022.

36 Lord Christopher Bellamy KC, s*upra* note 34; The Crown Prosecution Service, ‘Pay Ranges 2018/19 and 2019/2020’ (*CPS,* undated) <www.cps.gov.uk/sites/default/files/documents/publications/foi/2019/2019-foi-disclosure-24-attachment-A.pdf> accessed 26 June 2022.

37 House of Commons Justice Committee, *The Future of Legal Aid Third Report of Session 2021–22* (HC 70, 27 July 2021) https://committees.parliament.uk/publications/6979/documents/72829/default/ accessed 11 November 2022; The Westminster Commission on Legal Aid, *Inquiry Into the Sustainability and Recovery of the Legal Aid Sector* (The All-Party Parliamentary Group on Legal Aid 2021) <https://lapg.co.uk/wp-content/uploads/The-Westminster-Commission-on-Legal-Aid_WEB.pdf> accessed 19 January 2022.

38 James Thornton, ‘Is Publicly Funded Criminal Defence Sustainable? Legal Aid Cuts, Morale, Retention and Recruitment in the English Criminal Law Professions’ (2020) 40(2) Legal Studies 230 <http://dx.doi.org/10.1017/lst.2019.31> accessed 25 June 2023; Vicky Kemp, *Transforming Legal Aid: Access to Criminal Defence Services* (Legal Services Commission 2010) <https://core.ac.uk/download/pdf/33573065.pdf> accessed 16 April 2022.

39 Alisdair Gillespie and Siobhan Weare, *The English Legal System* (9th edn, Oxford University Press 2023), p. 351-2.

40 Catrina Denvir and Others, *supra* note 25.

41 Lord Christopher Bellamy KC, s*upra* note 34.

42 The Westminster Commission on Legal Aid, *supra* note 37; House of Commons Justice Committee, *supra* note 37.

43 Catrina Denvir and Others, *Legal Aid and the Future of Access to Justice* (Hart 2023) <https://eprints.gla.ac.uk/271785/1/271785.pdf> accessed 19 July 2023. p. 24.

44 Young Legal Aid Lawyers, 'Social Mobility in A Time of Austerity' (*Young Legal Aid Lawyers* 2018) http://www.younglegalaidlawyers.org/sites/default/files/Soc%20Mob%20Report%20-%20edited.pdf accessed 12 November 2021; Catrina Denvir and Others, *supra* note 25.

45 Barbri, ‘SQE1 Preparation’ *(Barbri,* undated) <www.barbri.com/sqe/sqe1-prep/> accessed 25 August 2024; Barbri, ‘SQE2 Preparation’ *(Barbri,* undated) <www.barbri.com/sqe/sqe2-prep/> accessed 25 August 2024; BPP, ‘Legal Practice Course (LPC)’ (*BPP,* undated) <www.bpp.com/courses/law/postgraduate/lpc-legal-practice-course?&?ppc_keyword=lpc%20courses&gclid=Cj0KCQjwuLShBhC_ARIsAFod4fKiqXgV2OS5cLlEvFgG3Khm75wq_aQM_AYc-r-XYR95PlG64aOquZEaAiBSEALw_wcB&gclsrc=aw.ds> accessed 25 August 2024; BPP, ‘SQE courses’ (*BPP*, undated) <www.bpp.com/courses/law/postgraduate/sqe-courses?www.bpp.com/courses/law/postgraduate/sqe-courses?gclsrc=aw.ds&?ppc_keyword=bpp%20sqe&gclid=CjwKCAiAxvGfBhB-EiwAMPakqiSOx5Z2bxh-ek2zHVbmf4oTSXQxch8KgiFG9ZpcNMABHMAsvhKrhxoC7agQAvD_BwE&gclsrc=aw.ds> accessed 25 August 2024; The College of Legal Practice, ‘Explore the SQE’ (*The College of Legal Practice*, undated) <www.collegalpractice.com/sqe> accessed 25 August 2024; Law Training Centre, ‘Course Fees’ (*Law Training Centre,* undated) <www.ltckent.co.uk/course-fees/#SQE1%20FEES> accessed 25 August 2024; UWE Bristol, ‘Solicitors Training Course (SQE Prep)’ (*UWE Bristol,* undated) <https://courses.uwe.ac.uk/M99F12/solicitors-training-course-sqe-prep#fees> accessed 25 August 2024; Nottingham Trent University, ‘SQE 1 Preparation Course PGDip’ (*Nottingham Trent University,* undated) <www.ntu.ac.uk/course/nottingham-law-school/pr/pgdip-sqe-1-preparation-course#entry-requirements> accessed 25 August 2024; Nottingham Trent University, ‘SQE 2 Preparation Course PGCert’ (*Nottingham Trent University,* undated) <<www.ntu.ac.uk/course/nottingham-law-school/pr/pgcert-sqe-2-preparation-course> accessed 25 August 2024; Nottingham Trent University, ‘LLM SQE 1 and SQE 2 Preparation Course PGCert’ (*Nottingham Trent University,* undated) <www.ntu.ac.uk/course/nottingham-law-school/pr/llm-sqe-1-and-sqe-2-preparation-course> accessed 25 August 2024; QLTS, ‘Setting Yourself Up to Pass The SQE with QLTS Prep Courses’ (*QLTS,* undated) <www.qlts.com/courses/> accessed 25 August 2024; Swansea University, ‘PGdip Legal Practice Course’ (*Swansea University*, undated) <www.swansea.ac.uk/postgraduate/taught/law/pgdip-legal-practice/> accessed 25 August 2024; Swansea University, ‘Your Guide to the Solicitors Qualifying Examination (SQE)’ (*Swansea University*, undated) <www.swansea.ac.uk/law/cplp/sqe-preparation/sqe-guide/?gclid=CjwKCAiAxvGfBhB-EiwAMPakqqhNNDO1DzVCrpxUrNkoz8No68h60d7sFztvSk4iqqw2GWHHOp2IRBoCCWcQAvD_BwE> accessed 25 August 2024; Swansea University, ‘Professional Legal Practice, LLM’ (*Swansea University*, undated) <www.swansea.ac.uk/postgraduate/taught/law/llm-professional-legal-practice/> accessed 25 August 2024; The University of Law, ‘Legal Practice Course (LPC)’ (*The University of Law*, undated) <www.law.ac.uk/study/postgraduate/law/lpc/> accessed 25 August 2024; University of Law, ‘SQE1 Preparation Course’ (*The University of Law*, undated) <www.law.ac.uk/study/postgraduate/law/sqe-1-preparation-course/> accessed 25 August 2024; University of Law, ‘SQE2 Preparation Course’ (*The University of Law*, undated) <www.law.ac.uk/study/postgraduate/law/sqe-2-preparation-course/> accessed 25 August 2024; The University of Law, ‘LLM Legal Practice (SQE1&2)’ (*The University of Law*, undated) <www.law.ac.uk/study/postgraduate/law/llm-sqe-1-and-2/> accessed 25 August 2024.

46 Independent Review of Criminal Legal Aid’ (*GOV.UK*, 21 December 2020) <www.gov.uk/government/groups/independent-review-of-criminal-legal-aid> accessed 29 April 2025.

47 Young Legal Aid Lawyers’ *supra* note 44; The Westminster Commission on Legal Aid, *supra* note 37.

48 ‘AGCAS First-Year Student Career Readiness Survey – Research Report’ (*The Association of Graduate Careers Advisory Services*, undated) <www.agcas.org.uk/knowledge-centre/b671b272-c0fe-40b4-85dd-1c19b529ddd6> accessed 3 June 2025; Jan Shury and others, ‘Planning for success: Graduates’ career planning and its effect on graduate outcomes’ (*Department of Education*, March 2017) <https://assets.publishing.service.gov.uk/media/5a7583c3e5274a1242c9ee85/Graduates__career_planning_and_its_effect_on_their_outcomes.pdf> accessed 12 June 2025; Kathleen M Quinlan and James Corbin, ‘How and why do students’ career interests change during higher education?’ [2023] Studies in Higher Education 1, <http://dx.doi.org/10.1080/03075079.2023.2166916> accessed 29 June 2025.

49 Lord Christopher Bellamy KC, s*upra* note 34; Catrina Denvir and Others, *supra* note 25.

50 Roxanna Dehaghani and Daniel Newman, ‘The Crisis in Legally Aided Criminal Defence in Wales: Bringing Wales Into Discussions of England and Wales’ (2021) 41(2) Legal Studies 234 <http://dx.doi.org/10.1017/lst.2020.42> accessed 15 June 2024; Daniel Newman and Roxanna Dehaghani, *‘Pressures of Practice: Introduction’* in Daniel Newman and Roxanna Dehaghani, Experiences of Criminal Justice: Perspectives From Wales on a System in Crisis (Policy Press 2022) <http://dx.doi.org/10.1332/policypress/9781529214222.003.0007> accessed 26 July 2023.

51 Ibid.

52 The Law Society, *supra* note 35.

53 The London Criminal Courts Solicitors’ Association, ‘Submission to The Independent Review of Criminal Legal Aid Call for Evidence’ (*LCCSA,* undated) <https://www.lccsa.org.uk/wp-content/uploads/2021/05/LCCSA-response-to-CLAR-Call-for-Evidence.pdf> accessed 8 February 2022.

54 Ibid.

55 Michelle Pauli, ‘Early Careers Survey 2021: Careers Advice and Guidance’ (September 2021) <https://luminate.prospects.ac.uk/early-careers-survey-2021-careers-advice-and-guidance#:~:text=The%20Prospects%20Early%20Careers%20Survey%202021%20has%20shown%20that%20significant,changes%20are%20also%20being%20transformed.> accessed 22 November 2023.

56 Lord Christopher Bellamy KC, s*upra* note 34.

57 John W Creswell and J David Creswell, *Research Design: Qualitative, Quantitative, and Mixed Methods Approaches* (4th edn, Sage Publications 2014).

58 Alan Bryman, *Social Research Methods* (Oxford University Press 2015).

59 Itamar Gati and Viktória Kulcsár, ‘Making Better Career Decisions: From Challenges to Opportunities’ (2021) 126 Journal of Vocational Behavior 103545, <http://dx.doi.org/10.1016/j.jvb.2021.103545> accessed 27 April 2022.

60 Chick, C, Pardo, S, Reyna, V, and Goldman, D, ‘Decision Making’ (2012) Encyclopedia of Human Behavior, (2), 651– 658 <https://doi.org/10.1016/B978-0-12-375000-6.00122-1> accessed 27 April 2022.

61 Robert West and Ashley Gould, ‘Improving Health and Wellbeing: A Guide to Using Behavioural Science in Policy and Practice’ (*Attention Required! | Cloudflare*, 2022) <https://phwwhocc.co.uk/wp-content/uploads/2022/11/A-Guide-to-Using-Behavioural-Science_ENGLISH.pdf> accessed 26 April 2025.

62 Icek Ajzen, 'The Theory of Planned Behavior' (1991) 50(2) Organizational Behavior and Human Decision Processes https://www.sciencedirect.com/science/article/abs/pii/074959789190020T> accessed 27 April 2022.

63 Stephen C Hickerson and Others, ‘Predicting Pharmacy Students’ Intention to Apply for a Residency: A Systematic Theory of Planned Behavior Approach’ (2017) 9(1) Currents in Pharmacy Teaching and Learning 12 <http://dx.doi.org/10.1016/j.cptl.2016.08.047> accessed 28 August 2023; Badingatus Solikhah, ‘An Application of Theory of Planned Behavior Towards CPA Career in Indonesia’ (2014) 164 Procedia - Social and Behavioral Sciences 397 <http://dx.doi.org/10.1016/j.sbspro.2014.11.094> accessed 28 August 2023.

64 Icek Ajzen*, supra* note 62.

65 Ibid.

66 Ibid.

67 Alphonsa Jose K and Surendra Kumar Sia, ‘Theory of Planned Behavior in Predicting the Construction of Eco-Friendly Houses’ [2022] 33(4) Management of Environmental Quality: An International Journal <http://dx.doi.org/10.1108/meq-10-2021-0249> accessed 25 August 2023.

68 V Carfora and Others, ‘Explaining Consumer Purchase Behavior for Organic Milk: Including Trust and Green Self-Identity Within the Theory of Planned Behavior’ (2019) 76 Food Quality and Preference <http://dx.doi.org/10.1016/j.foodqual.2019.03.006> accessed 26 August 2023.

69 Martin Fishbein and Icek Ajzen, *Predicting and Changing Behavior: The Reasoned Action Approach* (Taylor & Francis Group 2011.

70 Ibid.

71 Icek Ajzen*, supra* note 62.

72 Ibid.

73 Ibid.

74 Ibid.

75 Icek Ajzen and BL Driver, ‘Prediction of Leisure Participation from Behavioral, Normative, and Control Beliefs: An Application of the Theory of Planned Behavior’ (1991) 13(3) Leisure Sciences 185 <http://dx.doi.org/10.1080/01490409109513137> accessed 23 August 2023.

76 Isabelle Fort, Catherine Pacaud and Pierre-Yves Gilles, ‘Job Search Intention, Theory of Planned Behavior, Personality and Job Search Experience’ (2014) 15(1) International Journal for Educational and Vocational Guidance 57 <http://dx.doi.org/10.1007/s10775-014-9281-3> accessed 28 August 2023; Badingatus Solikhah, ‘An Application of Theory of Planned Behavior Towards CPA Career in Indonesia’ (2014) 164 Procedia - Social and Behavioral Sciences 397 <http://dx.doi.org/10.1016/j.sbspro.2014.11.094> accessed 28 August 2023.

77 Martin Fishbein and Icek Ajzen*, supra* note 69.

78 Ibid.

79 Icek Ajzen*, supra* note 62.

80 Martin Fishbein and Icek Ajzen*, supra* note 69.

81 Ibid.

82 Icek Ajzen*, supra* note 62.

83 Ibid.

84 Ibid.

85 Hio Wa Mak and J Mark Davis, ‘The Application of the Theory of Planned Behavior to Help-Seeking Intention in a Chinese Society’ (2013) 49(9) Social Psychiatry and Psychiatric Epidemiology 1501 <http://dx.doi.org/10.1007/s00127-013-0792-x> accessed 28 August 2023; Tariq Bhatti and Maizaitulaidawati Md Husin, ‘An Investigation of the Effect of Customer Beliefs on the Intention to Participate in Family Takaful Schemes’ (2019) 11(3) Journal of Islamic Marketing 709 <http://dx.doi.org/10.1108/jima-04-2018-0066> accessed 26 August 2023; Cecilia Perri, Carlo Giglio and Vincenzo Corvello, ‘Smart Users for Smart Technologies: Investigating the Intention to Adopt Smart Energy Consumption Behaviors’ (2020) 155 Technological Forecasting and Social Change 119991 <http://dx.doi.org/10.1016/j.techfore.2020.119991> accessed 26 August 2023.

86 Icek Ajzen, ‘Constructing A Theory of Planned Behavior Questionnaire’ (*Icek Ajzen,* 2019) <https://people.umass.edu/aizen/pdf/tpb.measurement.pdf> accessed 20 June 2022.

87 Ibid.

88 Ibid.

89 This author, in press

90 Ibid.

91 Martin Fishbein and Icek Ajzen*, supra* note 69.

92 Jill Francis and Others, ‘Constructing Questionnaires Based on the Theory of Planned Behavior: A Manual for Health Service Researchers’ [2004] Centre for Health Services Research, University of Newcastle upon Tyne <https://openaccess.city.ac.uk/id/eprint/1735/> accessed 20 May 2022.

93 Lynne M Connelly, ‘Pilot Studies’ (2008) 17(6) Medsurg Nurs <www.proquest.com/docview/230525260/fulltextPDF/FA4F6DE819434B56PQ/1?accountid=10792&sourcetype=Scholarly%20Journals> accessed 13 October 2023.

94 Daniel Soper, ‘Calculator: A-Priori Sample Size for Structural Equation Models’ (*Free Statistics Calculators*, undated) <www.danielsoper.com/statcalc/calculator.aspx?id=89> accessed 25 October 2023.

95 Joseph F Hair and Others, *A Primer on Partial Least Squares Structural Equation Modeling (PLS-SEM)* (2nd edn, Thousand Oaks: SAGE 2017) <www.pls-sem.net/pls-sem-books/a-primer-on-pls-sem-3rd-ed/> accessed 18 November 2023.

96 Daniel Soper, *supra* note 94.

97 Joseph F Hair and Others, *supra* note 95; Alan Bryman, *Social Research Methods* (Oxford University Press 2015).

98 Joseph F Hair and Others, *supra* note 95.

99 Joseph F Hair and Abdullah Alamer, ‘Partial Least Squares Structural Equation Modeling (PLS-SEM) in Second Language and Education Research: Guidelines Using an Applied Example’ (2022) 1(3) Research Methods in Applied Linguistics 100027 <http://dx.doi.org/10.1016/j.rmal.2022.100027> accessed 25 October 2023; Joseph F Hair and Others, *Partial Least Squares Structural Equation Modeling (PLS-SEM) Using R* (Springer International Publishing 2021) <http://dx.doi.org/10.1007/978-3-030-80519-7> accessed 1 October 2023.

100 Joseph F Hair and Other, ‘Partial Least Squares Structural Equation Modeling: Rigorous Applications, Better Results and Higher Acceptance’ (2013) 46(1-2) Long Range Planning 1 <http://dx.doi.org/10.1016/j.lrp.2013.01.001> accessed 15 October 2023; Joseph F Hair and Others, *supra* note 103; Joseph F Hair and Others, ‘Assessing Measurement Model Quality In PLS-SEM Using Confirmatory Composite Analysis’ (2020) 109 Journal of Business Research 101 <http://dx.doi.org/10.1016/j.jbusres.2019.11.069> accessed 15 October 2023.

101 Tanya N Beran and Claudio Violato, ‘Structural Equation Modeling in Medical Research: A Primer’ (2010) 3(1) BMC Research Notes <http://dx.doi.org/10.1186/1756-0500-3-267> accessed 26 October 2023; Indranarain Ramlall, *Definition of SEM, Applied Structural Equation Modelling for Researchers and Practitioners* (Emerald Publishing Limited 2016) <www-emerald-com.uoelibrary.idm.oclc.org/insight/content/doi/10.1108/978-1-78635-883-720161001/full/html> accessed 12 October 2023.

102 ‘SmartPLS’ (*SmartPLS 4*, undated) <www.smartpls.com> accessed 15 August 2023; Galit Shmueli and Others, ‘Predictive Model Assessment in PLS-SEM: Guidelines for Using PLSpredict’ (2019) 53(11) European Journal of Marketing 2322 <http://dx.doi.org/10.1108/ejm-02-2019-0189> accessed 25 July 2024; Nicole Franziska Richter and Ana Alina Tudoran, ‘Elevating Theoretical Insight and Predictive Accuracy in Business Research: Combining PLS-SEM and Selected Machine Learning Algorithms’ (2024) 173 Journal of Business Research 114453 <http://dx.doi.org/10.1016/j.jbusres.2023.114453> accessed 26 October 2023.

103 Joseph F Hair and Others, *supra* note 99; Joseph F Hair and Others, *supra* note 100.

104 Ibid.

105 Joseph F Hair and Others, *Advanced Issues in Partial Least Squares Structural Equation Modeling* (Sage Publications, 2017); Jörg Henseler and Others, ‘Common Beliefs and Reality About PLS: Comments on Rönkkö & Evermann (2013)’ (2014) 17(2) Organizational Research Methods 182 <http://dx.doi.org/10.1177/1094428114526928> accessed 10 October 2023; Joseph F Hair and Others, *supra* note 99.

106 Ibid; Joseph F Hair and Others, *supra* note 95.

107 Ibid.

108 Joseph F Hair and Others, *supra* note 100.

109 Ibid.

110 Jörg Henseler, Christian M Ringle and Marko Sarstedt, ‘A New Criterion for Assessing Discriminant Validity in Variance-Based Structural Equation Modeling’ (2014) 43(1) Journal of the Academy of Marketing Science 115 <http://dx.doi.org/10.1007/s11747-014-0403-8> accessed 14 October 2023.

111 George Franke and Marko Sarstedt, ‘Heuristics Versus Statistics in Discriminant Validity Testing: A Comparison of Four Procedures’ (2019) 29(3) Internet Research 430 <http://dx.doi.org/10.1108/intr-12-2017-0515> accessed 14 October 2023; Joseph F Hair and Others, *supra* note 103; Sandra Streukens and Sara Leroi-Werelds, ‘Bootstrapping and PLS-SEM: A Step-by-Step Guide to Get More Out of Your Bootstrap Results’ (2016) 34(6) European Management Journal 618 <http://dx.doi.org/10.1016/j.emj.2016.06.003> accessed 15 October 2023; Joseph F Hair and Others, *supra* note 103.

112 Joseph F Hair and Others, *supra* note; Joseph F Hair and Others, *supra* note 100. Joseph F Hair and Others, *supra* note 95.

113 Joseph F Hair and Others, *supra* note 99.

114 Ibid.

115 Ibid.

116 Marko Sarstedt and Others, ‘Partial Least Squares Structural Equation Modeling (PLS-SEM): A Useful Tool for Family Business Researchers’ (2014) 5(1) Journal of Family Business Strategy 105 <http://dx.doi.org/10.1016/j.jfbs.2014.01.002> accessed 31 October 2023.

117 Erik Mooi and Marko Sarstedt, *A Concise Guide to Market Research: The Process, Data, and Methods Using IBM SPSS Statistics* (3rd ed, Springer 2019); Joseph F Hair and Others, *supra* note 99.

118 Joseph F Hair and Others, *supra* note 99.

119 Ibid.

120 Ibid; Randall E Schumacker and Richard G Lomax, *A Beginner's Guide to Structural Equation Modeling* (3rd edn, Routledge 2010).

121 Ibid.

122 Ibid.

123 Ibid.

124 Ibid; Jacob Cohen, *Statistical Power Analysis for the Behavioral Sciences* (2nd edn, Routledge 1988).

125 Galit Shmueli and Others, ‘The Elephant in the Room: Predictive Performance of PLS Models’ (2016) 69(10) Journal of Business Research 4552 <http://dx.doi.org/10.1016/j.jbusres.2016.03.049> accessed 25 October 2023; Joseph F Hair and Others *supra* note 104; Joseph F Hair and Others, *supra* note 103.

126 Joseph F Hair and Others, *supra* note 99.

127 Ibid.

128 Amanda E Legate and Others, ‘PLS‐SEM : Prediction‐oriented Solutions for HRD Researchers’ [2021] 34(1) Human Resource Development Quarterly <http://dx.doi.org/10.1002/hrdq.21466> accessed 26 October 2023; Joseph F Hair and Others, ‘Partial Least Squares Structural Equation Modeling (PLS-SEM): An Emerging Tool in Business Research’ (2014) 26(2) European Business Review 106 <http://dx.doi.org/10.1108/ebr-10-2013-0128> accessed 22 September 2023.

129 Ibid.

130 Ibid.

131 NHS England, ‘Tens of Thousands of People Sign Up to Careers in the NHS’ (*NHS England*, 29 August 2022) <www.england.nhs.uk/2022/08/tens-of-thousands-of-people-sign-up-to-careers-in-the-nhs/> accessed 30 September 2024.

132 Darren Weir, ‘Future of Criminal Law Needs to Start with Students’ (*Law Gazette*, 30 July 2024) <www.lawgazette.co.uk/commentary-and-opinion/future-of-criminal-law-needs-to-start-with-students/5120502.article> accessed 16 August 2024.

133 Icek Ajzen*, supra* note 62.

